# Supplementary material for: The genome sequence of the September Thorn moth, Ennomos erosaria (Denis & Schiffermüller), 1775
Source: Wellcome Open Res. 2024 May 22;9:287. [Version 1] doi: 10.12688/wellcomeopenres.22254.1 (PMC12583911; doi:10.12688/wellcomeopenres.22254.1)
Supplement: Peer review history

# Reviewer report: Reviewer response for version 1

**DOI:** 10.21956/wellcomeopenres.24531.r126595

**Published:** 2025-11-03

**Authors:** Jason Charamis

**Affiliations:** 1 Foundation for Research and Technology - Hellas, Irákleion, Greece

This genome assembly report presents a high-quality chromosomal-level sequence of the endangered September Thorn moth ( *Ennomos erosaria*) from the UK's Darwin Tree of Life project. The assembly spans 423.1 Mb across 32 chromosomes including Z and W sex chromosomes, with excellent technical metrics (scaffold N50: 14.6 Mb, BUSCO completeness: 98.7%, QV: 60.4). Using PacBio HiFi sequencing and Hi-C scaffolding, researchers achieved 99.65% chromosomal assignment. Given the species' 87% population decline over 40 years, this genomic resource provides crucial foundation data for conservation genetics, evolutionary studies, and understanding this Geometridae family member's biology.

Are sufficient details of methods and materials provided to allow replication by others?

Yes

Is the rationale for creating the dataset(s) clearly described?

Yes

Are the datasets clearly presented in a useable and accessible format?

Yes

Are the protocols appropriate and is the work technically sound?

Yes

Reviewer Expertise:

Arthropod Comparative Genomics

I confirm that I have read this submission and believe that I have an appropriate level of expertise to confirm that it is of an acceptable scientific standard.

# Reviewer report: Reviewer response for version 1

**DOI:** 10.21956/wellcomeopenres.24531.r99542

**Published:** 2024-10-01

**Authors:** James Mallet

**Affiliations:** 1 Harvard University, Cambridge, Massachusetts, USA

This is another useful genome assembly to add to the many other British species already carried out in the Darwin Tree of Life series. The chromosomal level assembly seems well done from the reported coverage and BUSCO scores etc. 

 I have no other particularly useful comments.

 I noticed one phrase that could be reworded: "the density of the BUSCO genes from the closest taxonomically lineage" -- I would say "...from the taxonomically closest lineage".

Are sufficient details of methods and materials provided to allow replication by others?

Yes

Is the rationale for creating the dataset(s) clearly described?

Yes

Are the datasets clearly presented in a useable and accessible format?

Yes

Are the protocols appropriate and is the work technically sound?

Yes

Reviewer Expertise:

Lepidoptera genetics and evolution

I confirm that I have read this submission and believe that I have an appropriate level of expertise to confirm that it is of an acceptable scientific standard.
